# SQM-LRU: A Harmony Dual-Queue Management Algorithm to Control Non-Responsive LTF Flow and Achieve Service Differentiation

**DOI:** 10.3390/s21103568

**Published:** 2021-05-20

**Authors:** Penghui Li, Xianliang Jiang, Jiahua Zhu, Guang Jin

**Affiliations:** Faculty of Electrical Engineering and Computer, Ningbo University, 818 Fenghua Road, Ningbo 315211, China; 1911082059@nbu.edu.cn (P.L.); 1811082205@nbu.edu.cn (J.Z.); jinguang@nbu.edu.cn (G.J.)

**Keywords:** non-responsive LTF flow, responsive flow, dual-queue management, shadow queue, improved LRU, service differentiation, AQM

## Abstract

The increase in network applications diversity and different service quality requirements lead to service differentiation, making it more important than ever. In Wide Area Network (WAN), the non-responsive Long-Term Fast (LTF) flows are the main contributors to network congestion. Therefore, detecting and suppressing non-responsive LTF flows represent one of the key points for providing data transmission with controllable delay and service differentiation. However, the existing single-queue management algorithms are designed to serve only a small number of applications with similar requirements (low latency, high throughput, etc.). The lack of mechanisms to distinguish different traffic makes it difficult to implement differentiated services. This paper proposes an active queue management scheme, namely, SQM-LRU, which realizes service differentiation based on Shadow Queue (SQ) and improved Least-Recently-Used (LRU) strategy. The algorithm consists of three essential components: First, the flow detection module is based on the SQ and improved LRU. This module is used to detect non-responsive LTF flows. Second, different flows will be put into corresponding high or low priority sub-queues depending on the flow detection results. Third, the dual-queue adopts CoDel and RED, respectively, to manage packets. SQM-LRU intends to satisfy the stringent delay requirements of responsive flow while maximizing the throughput of non-responsive LTF flow. Our simulation results show that SQM-LRU outperforms traditional solutions with significant improvement in flow detection and reduces the delay, jitter, and Flow Completion Time (FCT) of responsive flow. As a result, it reduced the FCT by up to 50% and attained 95% of the link utilization. Additionally, the low overhead and the operations incur O(1) cost per packet, making it practical for the real network.

## 1. Introduction

With the emerging applications such as virtual reality, video conferencing, real-time online gaming, etc., the requirements for latency, jitter, and throughput have reached a new level. These new requirements indicate that there is a demand for new designs for the network protocols. The early queue management algorithms, such as RED [1], CoDel [2], etc., were built to mainly serve a small range of applications with identical needs primarily based on a throughput-oriented design. It also did not incorporate mechanisms to distinguish different traffic. Since the beginning of this year, there has been an increase in the type of Internet transfer. However, there was not any significant improvement made on the queue management algorithm to properly support the growing network traffic’s complex demands. This raises the question of “How to meet differentiated service quality” in order to overcome the critical challenge seen by Internet networks today.

The detection and control of LTF flow have attracted much attention [3,4,5,6,7]. The non-responsive LTF flow accounted for most network traffic and is the main contributor to network congestion, and there is only a limited performance gain by dropping packets from the responsive flow. However, scheduling packets preferentially from the non-responsive LTF flow can significantly enhance the Quality of Service (QoS) [8]. Therefore, the detection and control of the non-responsive LTF flow can effectively protect the responsive flow from the lack of resources and improve network transmission’s performance.

**Figure 1:**
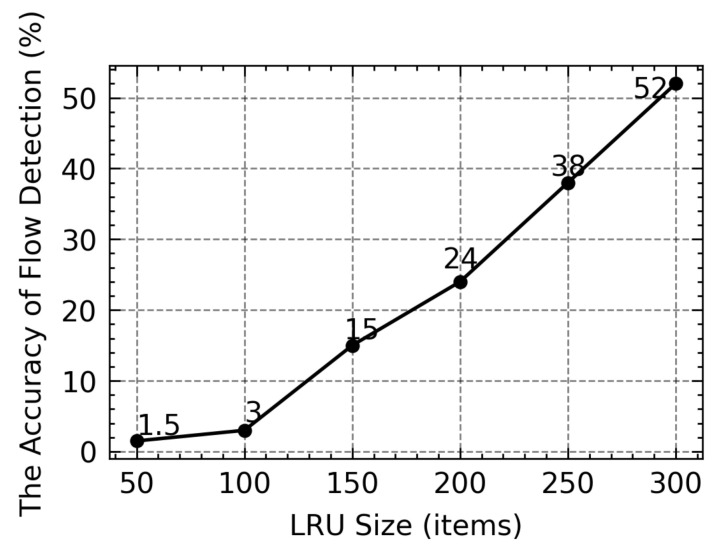
The influence of Least-Recently-Used (LRU) size on accuracy.

**Figure 2:**
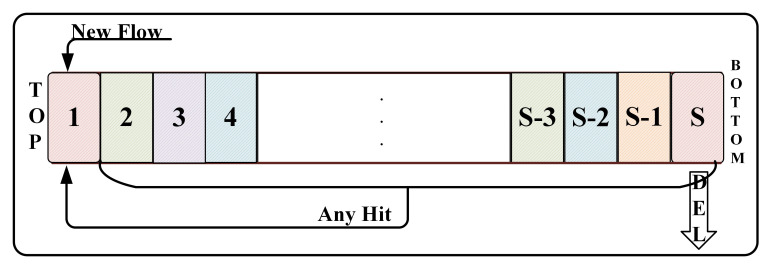
The LRU concept map.

The LRU caching scheme [6] with a partial flow state to detect the non-responsive LTF flow and allots fixed size cache to record recent flows by constantly bringing the incoming flow to the top of the cache and replacing the least hit flow with the newly sampled flow. Each flow maintains a packet counter to identify the non-responsive LTF flow and updates the counter as each packet enters. Only when the counter exceeds the pre-defined threshold, the flow is reported as non-responsive LTF flow. Although it works well when the cache size is large, the performance degrades when the cache becomes smaller. Figure 1 shows the accuracy of flow detection in different LRU size. A large-size cache is required to achieve higher flow detection. This is due to the excessive number of Short-Lived or Slow (SLS) flows, which results in frequent removal of LTF flows to make room for SLS flows so that they will be added into LRU. More real, non-responsive LTF flows are not reported correctly as they can be expelled from the cache before the count exceeds the threshold. Similarly, the malicious on/off flows will be flushed out of the cache as soon as it stops sending packets, pushing the intermittent packets and easily escaping the LRU. Intuitively, this problem can be addressed by simply increasing the cache size. However, this scheme requires searching and updating the cache at the order of line speed for each incoming packet. The implementation of the LRU cache requires costly high-speed static Random Access Memory (RAM) or Content Addressable Memory (CAM). Therefore, adopting a large-size cache can cause scalability concern for the LRU scheme to be deployed at high-speed routers. The LRU-based scheme principle can be seen in Figure 2, in which the new flow is inserted and updated at the TOP node. When the cache is full, the longest non-hit flow record will be removed.

Motivated by above issues, we propose a novel dual-queue active queue management algorithm (SQM-LRU) based on the SQ and Mark-LRU (M-LRU). Our main contributions are as follows:

- First, the SQM-LRU is presampled in the SQ, then for further comparison and update in the M-LRU. The M-LRU includes a Mark node to separate the non-responsive LTF and responsive flow. The new flow and flow with the counter below the threshold will be placed at the Mark location. Furthermore, it can use less space to achieve a higher flow detection accuracy, and the operations incur O(1) cost per packet.
- Then, by placing packets detected as the non-responsive LTF flow into the low-priority sub-queue and others into the high-priority sub-queue to contain and penalize non-responsive LTF flow to achieve fairness when responsive flow coexists with non-responsive LTF flow.
- In addition, the dual-queue adopts the improved RED and CoDel for active queue management coupled with the method above. In this way, it enables low delay for responsive flow while maximizing the throughput for non-responsive LTF flow.

The rest of the paper is organized as follows. Section 1 provides a brief review of the related work. Section 2 explains the design of SQM-LRU and its components. Section 3 gives the detailed performance evaluation and discussions, and Section 4 concludes this paper.

## 2. Related Work

Today, the traffic on the Internet tends to be fluctuating and demanding. Thereby, the queue management algorithm is designed to tame overflowing traffic and penalize flows that persistently overuse bandwidth. There are two categories of queue management algorithms: Passive Queue Management (PQM) and Active Queue Management (AQM) [9]. In PQM, the router discards packets if the queue is full. Traditional Internet routers employ DropTail for managing the queue. It simply sets a maximum length for the queue, and it enqueues packets until the maximum length is reached, then drops subsequent incoming packets until the queue is decreased below its maximum value. DropTail allows the router to maintain high queue occupancy, but it tends to discriminate against bursty traffic and drops many packets while producing global synchronization of sources. However, with DropTail, the decision to drop a packet is essentially a passive way, and it has several flaws that prompted research into a more active approach to router queue management. These flaws are most apparent during periods of persistent congestion and include lock-out and full queues. When network congestion occurs, it only simply treat congestion and can not avoid congestion occurrence. Besides, it does not distinguish between UDP and TCP flows. Moreover, the TCP flow is disadvantageous in resources competing, not having a fair guarantee. Approaches like Random Drop or Drop Front are similar to DropTail and do not solve the problem above. The only difference they have from DropTail is the criteria used to choose which packet to discard when the queue get full. The Random Drop randomly chooses a packet in the queue, and the latter drops the packet at the front of the queue. In practice, most routers use the simplistic DropTail algorithm, which is simple to implement with minimal computational overhead. The solution to the above problem is for routers to drop packets before a queue becomes full so that that end nodes can respond to congestion before buffers overflow. Such a proactive approach is called AQM, which by dropping/marking packets before network congestion, so that end nodes can respond to packet losses and reduce the sending rates.

Random Early Detection (RED) is a typical AQM algorithm designed to minimize packet loss and maintain high link utilization. RED evaluates the changes in network congestion by calculating the average queue length. When the average queue length increases rapidly, the probability of packet drop increases, notifying the sender to decrease their packet sending rate appropriately and avoid network congestion. However, RED is sensitive to the parameter settings, a sudden increase in the packet drop probability may occur when the average queue length is greater than the queue length upper limit. FXRED, presented by Adamu et al. [10], recognizes the state of the current network’s traffic load and auto-tunes its drop probability suitable to the observed load situation to maintain stable and better performance. Su et al. [11] use the Q-learning algorithm to achieve self-adaptive adjustment for the $max_{p}$ of RED. The QRED algorithm avoids the sensitivity of the RED algorithm parameters and adapts the packet loss rate according to the specific network service type. To meet the strict delay requirements, researchers have proposed low-latency AQM such as CoDel (Controlled Delay Management) [2] and PIE (Proportional Integral controller-Enhanced) [12]. CoDel directly controls the router/host queuing delay. It timestamps packets as they are enqueued. If the sojourn time spent by a packet within the queue is higher than a predefined threshold, the algorithm sets a timer to drop a packet at dequeue and indirectly signals the end-hosts to adjust the packet sending rates for maintaining the low delay when the burst traffic arrives. An alternate AQM scheme, Proportional Integral controller Enhanced (PIE), was also proposed to ensure the transmission of delay-sensitive flow. The Minstrel PIE proposed [13] can significantly improve traffic performance when there are multiple traffic bottlenecks. Sadek et al. [14] present the design of a PID controller based on state feedback. The PID parameters are computed using the Linear Matrix Inequality (LMI) technique and were applied to AQM at the router to avoid congestion.

Furthermore, the above scheme avoids congestion by dropping/marking packets. Different schemes use feedback to avoid congestion efficiently, and various schemes use Explicit Congestion Notification (ECN) [15]. The ECN allows end-to-end congestion notification between two end-hosts on TCP/IP-based networks, where ECN must be enabled on both end-hosts and all intermediate devices. Typical ECN schemes [16,17,18,19] have shown that ECN schemes help increase throughput and reduce delay.

Meanwhile, some LRU-based schemes have been proposed to distinguish flows, such as LRU-DCBF [20], hierarchical LRU [21], etc. These schemes perform flow recording and updating by constructing the LRU cache. However, when storage is limited, all suffer from performance degradation. Besides, Li et al. [22] proposed low-rate flow elimination and d-Left hash table for flow detection.

Most of the above schemes were designed to serve only a small number of applications with similar needs (achieving only low-latency or high-throughput), which hardly achieve differentiated services. When the responsive flow coexists with non-responsive LTF flow, they have different bandwidth-grabbing capabilities, and the performance of responsive flow is is significantly impaired. Besides, these schemes cannot meet the different flows’ QoS requirements. In order to tackle above issues, we propose the SQM-LRU. It adopts SQ and M-LRU to detect the non-responsive LTF flow. Based on the flow detection results, the priority dual-queue is used to ensure transmission demand and achieve fairness. The dual-queue adopts CoDel and RED to achieve low delay for responsive flow while maximizing the throughput of non-responsive LTF flow. The low overhead involved and the operations incur O(1) cost per packet, making it feasible in current high-speed routers and data centers.

## 3. Proposed SQM-LRU Scheme

### 3.1. Motivation

The response to network congestion distinguishes responsive and unresponsive flows. When network congestion occurs, the responsive flow actively reduces the packet sending rates. Flows based on Transmission Control Protocol (TCP) protocol, such as Telnet, FTP and HTTP, are typical responsive traffics [23]. In this paper, flows generated by interactive applications, such as Unreliable Datagram Protocol (UDP) with lower sending rates, are unified into the responsive flow. A typical unresponsive flow is generated by the unresponsive protocol such as UDP. This flow is also known as malicious or selfish flow. During network congestion, it does not reduce the packet sending rates, which further exacerbates network congestion. UDP-based flow does not have a built-in congestion control protocol, and it cannot react to network congestion. In this paper, UDP with high sending rates is classified as the non-responsive LTF flow.

**Figure 3:**
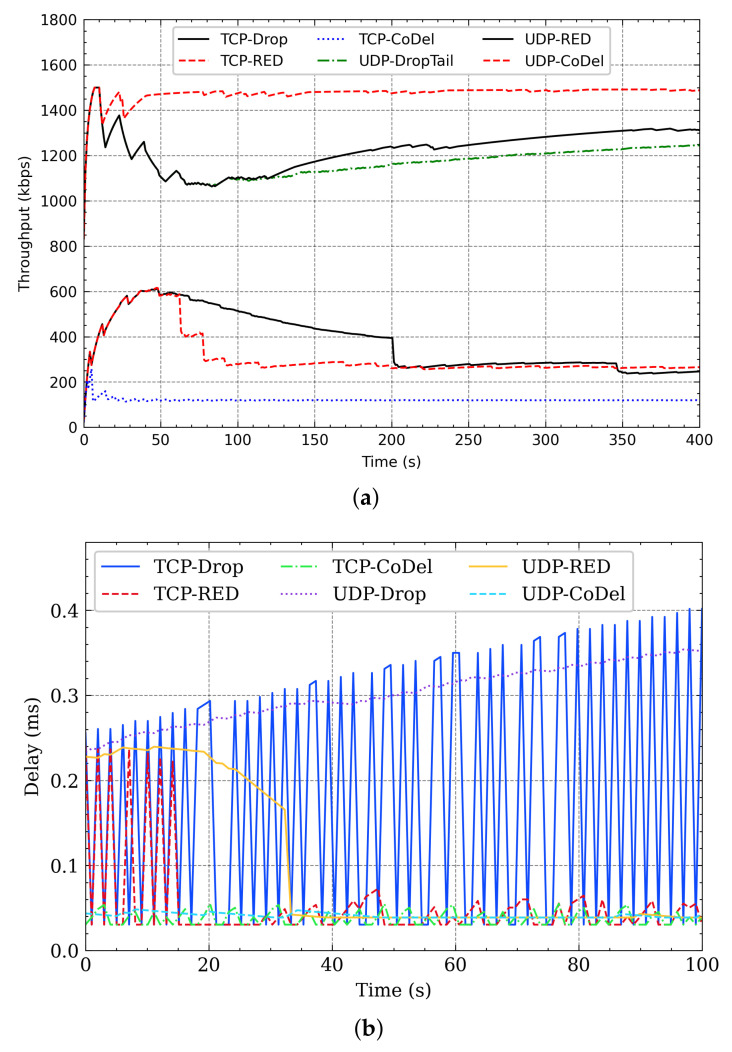
TCP and UDP coexist. (**a**) The throughput when TCP and UDP coexist. (**b**) The delay when TCP and UDP coexist.

When the responsive flow coexists with non-responsive LTF, as DropTail, RED, and CoDel are unable to distinguish flow, which leads to poor fairness, also, these queue management algorithms are only designed to serve a single quality of service and cannot provide differentiated service requirements for different flows. It can be seen from Figure 3 that the throughput and delay of TCP (responsive flow) cannot be guaranteed when it coexists with UDP (non-responsive LTF flow) under DropTail (PQM), RED, and CoDel (AQM). Therefore, the effective distinction between different flows helps to achieve fairness. Based on fairness, the responsive flow is more sensitive to delay and jitter. The non-responsive LTF flow tends to obtain higher throughput as soon. Therefore, assigning differentiated queue management algorithms for different flows could meet their QoS requirements.

### 3.2. SQM-LRU’s Design

**Figure 4:**
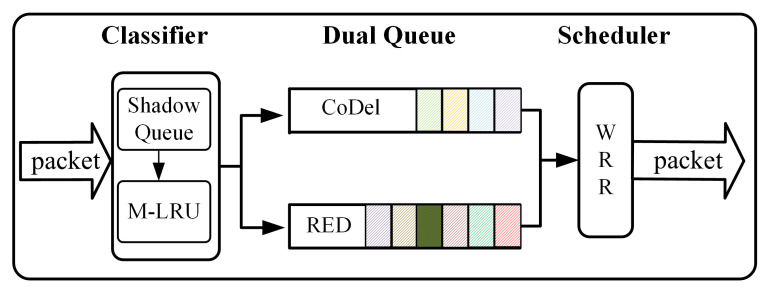
The big picture of SQM-LRU’s design.

The big picture of the SQM-LRU is shown in Figure 4. It mainly consists of three components: classifier, dual queue, and scheduler. The classifier adopts SQ and M-LRU to detect the non-responsive LTF flow and figure out the flow’s current condition. In the following sections, the features of each block in Figure 4 will be explained in detail.

**Figure 5:**
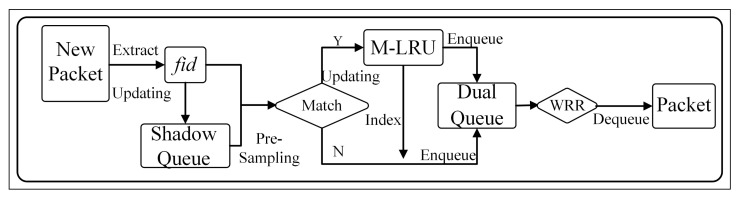
The SQM-LRU’s algorithm flow-chart.

The flowchart is shown in Figure 5. The $f_{id}$ is the globally unique value of the packet’s quadruple information (source IP, destination IP, source port, and destination port) via the hash function. Packets have the same $f_{id}$ as belongs to the same flow. When a new packet enqueues, the $f_{id}$ is first obtained and pre-sample with probability $p_{samp}$ in SQ. Furthermore, the sampling result is matched with the $f_{id}$. Then, according to the matched outcome, further updates in the M-LRU to detect whether the packet belongs to the non-responsive LTF flow. Based on the detection results, the packets are placed into different priority sub-queues. After, the WRR scheduling algorithm is used to schedule between high- and low-priority sub-queues when dequeuing packets.

### 3.3. Pre-Sampling in SQ

The SQ records the historical packet information by constructing a copy of the packet that flows through the route. Moreover, it maintains a fixed-length queue. Its length is larger than the physical queue in route. Therefore, it can record more historical packet information as soon. Moreover, it only records the $f_{id}$ of the packet, so it takes less additional space. SQ reflects the packet distribution, and the probability of each flow being sampled is shown in Equation (1), where $b_{n}$ represents the number of packets belonging to flow *n* in the SQ. With the high packet sending rate flow with the more frequently packet distributed in the SQ and the flow with a lower sending rate or actively reduces the sending rate, the distribution is more sparse. By analyzing the distribution of packets in the SQ, we can identify the type of flow preliminarily.

(1) $$p_{\mathrm{pick}\;}=p_{\mathrm{samp}\;}\frac{b_{n}}{SQ}$$

**Figure 6:**
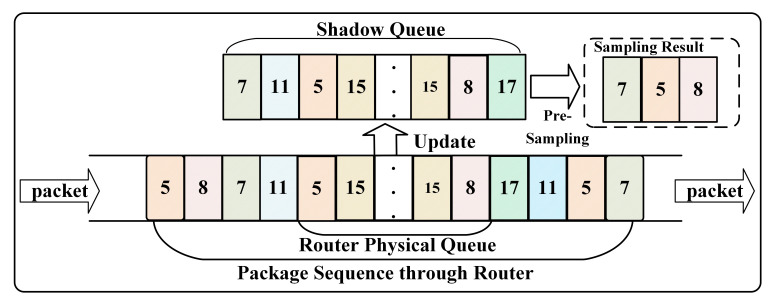
The SQ’s principle.

Figure 6 shows a pictorial example of how the SQ functions. When a new packet enters the router, first, the SQM-LRU gets the $f_{id}$ of the new packet, and then pre-sampling with a setting probability $p_{samp}$ in SQ. In addition, SQM-LRU matching the sampling results with a new package in $f_{id}$ for further updating in the M-LRU. Furthermore, the SQ is updated with the new packet’s $f_{id}$. The larger the $p_{samp}$, the smaller the sampling error, and the more it can reflect packets’ distribution. However, a larger $p_{samp}$ may cause a heavy computational burden, so it is necessary to select an appropriate $p_{samp}$ to balance efficiency.

### 3.4. The M-LRU

**Figure 7:**
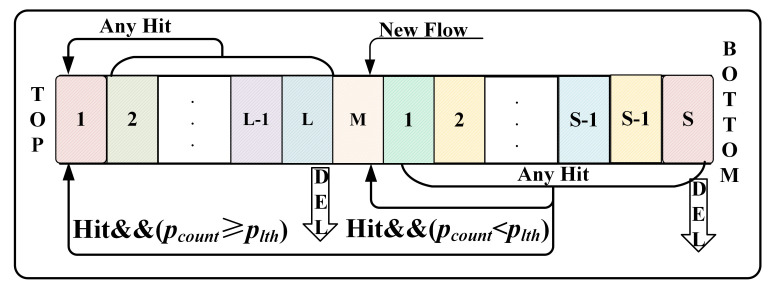
Schematic diagram of M-LRU.

As shown in Figure 7, the M-LRU contains three empty nodes: TOP, MARK, and BOTTOM. It differs from LRU in the following aspects: (a) Only packets that are successfully matched in the SQ and then updated in M-LRU based on the $p_{info}$. (b) After updating the packet counter $p_{count}$, if it below the threshold $p_{lth}$, this item will be moved to after MARK node. Otherwise, it is moved to the TOP node. If the $p_{info}$ does not exist in M-LRU, it will be inserted into the MARK node and initialize its $p_{count}$ to 0. (c) If the M-LRU is full, the MARK or BOTTOM node will be deleted, i.e., the longest responsive or non-responsive LTF flow that has not appeared.

The traditional LRU scheme will update and insert all only at the TOP node (as shown in Figure 2). However, in SQM-LRU, the insertion is performed at the MARK node, and only the packet counter reaches the threshold $p_{lth}$ are brought to the TOP node. This way effectively addresses the non-responsive LTF flow frequently replaced by a large number of SLS flows in the LRU and effectively enhances the flow detection efficiency.

### 3.5. The Classifier Combined of SQ and M-LRU

**Figure 8:**
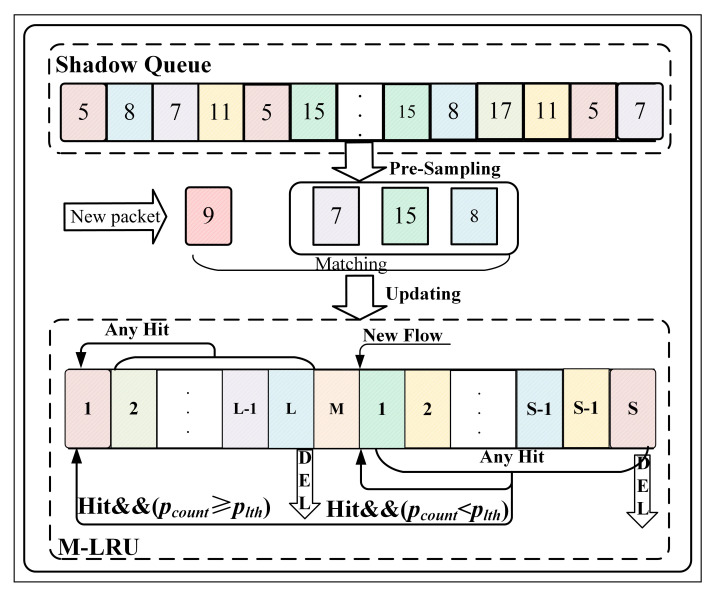
Combine SQ and M-LRU for flow detection.

Figure 8 shows the combination of the SQ and M-LRU for efficient flow detection. First, when a new packet enqueues, it is pre-sampled in the SQ before further comparison and updates in the M-LRU. Then, it matches the sampling result with the new packet. If the match is successful, it needs to be updated in M-LRU according to the $p_{info}$. If unmatched, it indicates the packet in the route is infrequent, and then it will be judged whether it belongs to the non-responsive LTF flow, which is already recorded in the M-LRU. If it is, the corresponding record in the M-LRU will be brought to the TOP node. The use of SQ and M-LRU reduces the space requirements for deployment in high-speed routing and prevents the non-responsive LTF flow from being frequently replaced with guaranteed flow detection efficiency.

The SQ is implemented as a queue, and a double-linked list is used for the M-LRU and its insertion, deletion of flow cost O(1) time. As a doubly-linked list, searching would take linear time, and instead a hash table is used to make search O(1). When a new item is added to the M-LRU, a corresponding entry is made in the hash table so that the search would take O(1) time. The memory cost is proportional to the size “S” of the M-LRU cache and the SQ, and the double-linked list includes three empty nodes: Top, Mark, and Bottom. In which the Mark node separates the non-responsive LTF flow and the responsive flow units. Algorithm 1 describes the classifier combining with SQ and M-LRU. $p_{count}$ is the number of this packet successfully matched in M-LRU.
**Algorithm 1:** The Classifier algorithm combining with SQ and M-LRU. **input**: The Packets Sequence **output**: The type of Packets **_1_ Initialize:** $p_{count}=0;$ $p_{lth}=5;$ pkt_num=0; M−LRU_size=200; pkt_num=0; $p_{samp}=0.4.$ **_2_ for** *Packet← Packets* **do** **_3_**  (pkt_info ← new Packet; **_4_**  pkts_samp = PreSampling (SQ,$p_{samp}$); **_5_**  **if** *pkt_info ∈ pkts_samp* **then**( **_6_**   **if** *pkt_info ∈ M-LRU* **then**( **_7_**    $p_{count}\;$ += 1; **_8_**    **if** *($p_{count}>p_{lth}$)&&(pkt_info_type==ResponsiveFlow)* **then**( **_9_**    MoveToTOP pkt_info; **_10_**    pkt_info_type=Non-ResponsiveLTF; **_11_**    Non-ResponsiveLTFcount+= 1; **_12_**    ResponsiveFlowcount−= 1; **_13_**    **if** *Non-Responsive LTF count>Non-Responsive LTF Unit Size* **then**( **_14_**    Drop LRU-MARK; **_15_**    **else if** *($p_{count}>p_{lth}$)&&(pkt_info_type==Non−ResponsiveLTF)* **then**( **_16_**    MoveToTOP (*pkt_info*); **_17_**    **else**( **_18_**     MoveToMARK (*pkt_info*); **_19_**  **else**( **_20_**   InsertToMARK (*pkt_info*); **_21_**   pkt_info_type = Non-ResponsiveLTF; **_22_**   ResponsiveFlowcount += 1; **_23_**   $p_{count}\;=\;1$; **_24_**   **if** *Responsive Flow count > Responsive Flow Unit Size* **then**( **_25_**    Drop (*LRU-BOTTOM*); **_26_**  pkt_num += 1; **_27_**  SQ[pkt_num]← pkt_info;

### 3.6. Dual-Queue

This paper adopts the priority dual-queue to achieve fairness while meeting the flow differentiated performance requirements. The dual-queue includes the high and low priority sub-queues. When a packet enters the router, the SQ and M-LRU are used to detect the flow. According to the detection result, if the packet belongs to the responsive flow, it will be put into the high priority sub-queue. Otherwise, it will be placed into the low priority sub-queue. The priority queue could achieve fairness when responsive flow coexists with non-responsive LTF flow. Furthermore, the high priority sub-queue adopts the CoDel to reduce the delay and jitter of responsive flow. The RED is used for low-priority sub-queues to maximize the bandwidth of non-responsive LTF flow. Each sub-queue adopts differentiated queue management schemes to meet various QoS of flows. When the packet is leaving, a scheduling algorithm is used to schedule packets between the high and low priority sub-queues.

RED [24] adds some new mechanisms compared to the DropTail: (a) minimizing packet loss and queuing delay, (b) avoiding global synchronization of sources, (c) maintaining high link utilization, and (d) removing biases against bursty sources. RED drops packets with a certain probability by computing the average queue length, expressed as Equation (2), where $w_{q}\in$ [0,1] is the weight equivalent to the low pass filter time constant, and *q* is the current queue length. The dropping probability can be calculated as Equation (3). RED can achieve high throughput in large-volume data transmission. However, for delay-sensitive applications, such as audio, control, Telnet, etc., RED may result in packet loss and high delay. CoDel [4] was developed to minimize delay by packets in the running buffer window. The aim is to keep the delay below 5 milliseconds. If the minimum delay rises too high, packets are dropped from the queue until the delay drops below the maximum level. Therefore, in this paper, we adopt CoDel for high priority sub-queue and RED for low priority sub-queue based on the above analysis. The use of RED and CoDel for the priority sub-queue will satisfy the stringent delay requirements of responsive flow while maximizing non-responsive LTF flow throughput. The impact of different sub-queue management algorithms will be detailed in Section 3.

(2) $$avg=\left\{\begin{array}{cc} \left(1-w_{q}\right)\times avg+w_{q}\times q_{1}\times q, & \;\mathrm{if}\;q<0\\ \left(1-w_{q}\right)\times avg, & \;\mathrm{otherwise}\; \end{array}\right.$$

(3) $$p_{b}=\left\{\begin{array}{cc} 0, & \;\mathrm{avg}\;\in \left[0,min_{th}\right)\\ \frac{\max_{p}\left(\mathrm{avg}-min_{th}\right)}{\max_{th}-min_{th}}, & \mathrm{avg}\in \left[\min_{th},\max_{th}\right)\\ 1, & avg\in \left[\max_{th},+\infty \right] \end{array}\right.$$

The queue scheduling algorithm aims to control the sending order of packets in priority sub-queues and balance the output rate of each sub-queue to utilize the network link resources. The common queue scheduling algorithms include FIFO (First In First Out), WFQ (Weighted Fairness Queuing), RR (Round Robin), WRR (Weighted Round Robin), SP (Strict Priority), etc. The impact of queue scheduling algorithms will be briefly analyzed in Section 3. The dual-queue algorithm (Algorithm 2) can be summarized as follows:
**Algorithm 2:** The Dual-Queue Algorithm. **input**: The type of Packets **output**: The ordered Packets Sequence **_1_ Initialize:** Highsub-Queue = CoDel; Alg = WRR; Lowsub-Queue = RED. **_2_ for** *Packet← Packets* **do** **_3_**  (**if** *pkt_info_type == Responsive Flow* **then**( **_4_**  EnQueueToHigh (*Packet*); **_5_**  **else**( **_6_**    EnQueueToLow (*Packet*); **_7_** packet = Scheduling (*High sub-Queue, Low sub-Queue, Alg);*

## 4. Simulation Analysis

In this section, the NS-2 [25] was used to evaluate the performance of SQM-LRU. This section first introduces the experimental settings, topology, and evaluation metrics, followed by analyzing the effects of different numbers of parallel flows on throughput and other performance, then details the impact of parameter settings. The sub-queue management and queue scheduling algorithms are also involved.

### 4.1. Simulation Scene and Parameter Setting

**Figure 9:**
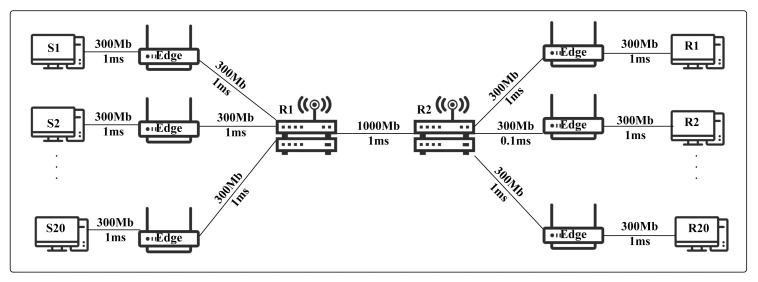
Network topology.

The simulation uses the typical single-bottleneck network topology, as shown in Figure 9. There are 20 senders (S1–S20), 20 receivers (R1–R20), and 2 core routers (R1 and R2) in the network. The link between each end-host and edge router has a bandwidth of 300 Mbps and a baseline delay of 1 ms. Between the two core routers R1 and R2, the bandwidth and base delay are 1000 Mbps and 1 ms, respectively. In the simulation, the standard parameters are set as follows: the senders have 180 flows transmitted in parallel, which includes 120 standard TCP (60 Telnet and 60 FTP flows) and 60 UDP flows. The Telnet and FTP randomly select the packet sending time and the flow size. The UDP flow pumping packet with a 3 Mbps rate and the CBR is selected as the traffic generator. Packets have a fixed size of 1000 bytes. Besides, we are also adding noise in the link to simulate the real network. The SQM-LRU algorithm in the implementation of the link between the two core routers. The other links perform the DropTail algorithm.

The high and the low priority sub-queue adopts CoDel and RED respectively. When the data packets are out of the queue, the WRR scheduling algorithm [26] is used. The hardware environment is as follows: the operating system is Ubuntu16.04, the processor is Intel(R) Core i5, the architecture is X86, system memory is 8G. The initial setup parameters are set as shown in Table 1.

For comparison, we, respectively, analyze the throughput, delay, jitter, packet loss rate, FCT, etc. We also use the confusion matrix to distinguish flow. The binary confusion matrix is defined in Table 2, and the accuracy is shown in Equation (4). The Positive Predictive Value (PPV) is defined as Equation (5) representing the non-responsive LTF flow that are correctly reported. True Negative Rate (TNR) measures the proportion of responsive flow correctly identified as defined in Equation (6).

(4) $$Acc=\frac{TP+TN}{TP+FN+TP+TN}\times 100\%$$

(5) $$PPV=\frac{TP}{TP+FP}\times 100\%$$

(6) $$TNR=\frac{TN}{TN+FN}\times 100\%$$

Fairness metrics are used in network engineering to determine whether flows are receiving a fair share of system resources. It can be measured by Jain’s Fair Index (JFI) [27], defined as Equation (7). The larger the JFI value, the better the fairness of this algorithm, where $x_{i}$ represents the throughput of flow *i*, and *N* represents the number of flows.

(7) $$JFI=\frac{{\left(\sum_{i=1}^{N}x_{i}\right)}^{2}}{N\sum_{i=1}^{N}x_{i}^{2}}$$

### 4.2. The Delay, Throughput and FCT

This group of simulations compares the throughput and other metrics of SQM-LRU with the traditional scheme. When the responsive and the non-responsive LTF flow coexist in bottleneck links, the non-responsive LTF flow (CBR) is more aggressive and occupies a wide bandwidth. Additionally, it does not respond to network congestion (reducing the sending rate), which seriously affects the transmission of the responsive flow. DropTail drops packets when the queue is full and unable to distinguish flow. RED also fails to achieve fairness of TCP with UDP flow. The TCP flow responds to packet discard, but the UDP flow does not, making UDP flow occupy much more bandwidth. Besides, RED does not integrate the priority mechanism, and it cannot adapt to the different QoS requirements. CoDel has been built and designed to solve the full buffer problem by limiting the packet queue delay and packet loss to enhancing the network’s overall performance. However, CoDel shows worse fairness comparing with RED. Therefore, the delay, jitter, and FCT of the responsive flow are significantly worse than the non-responsive LTF flow. The LRU and L-LRU have slightly improved performance but use a larger LRU cache space (500). The SQM-LRU detects the non-responsive LTF flow more precisely and puts these packets into the low priority sub-queue, and responsive flow packets are placed in a high priority sub-queue. As a result, the delay jitter, FCT of FTP and Telnet are significantly improved.

**Figure 10:**
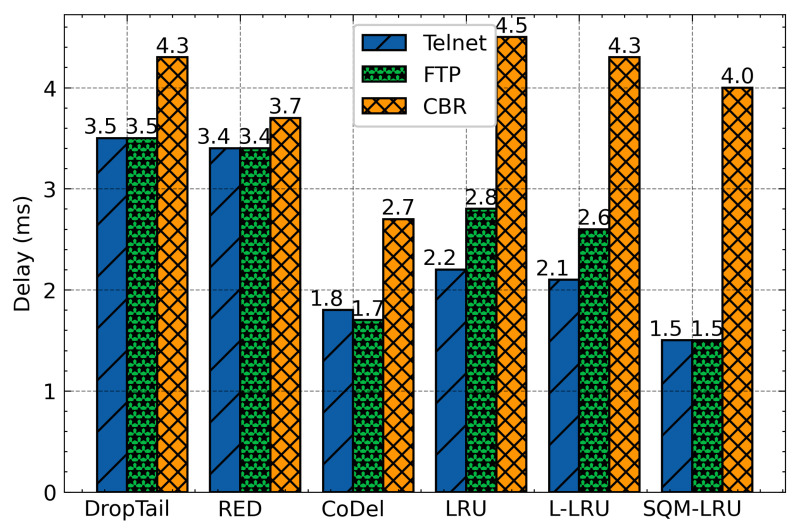
Average delay comparison.

**Figure 11:**
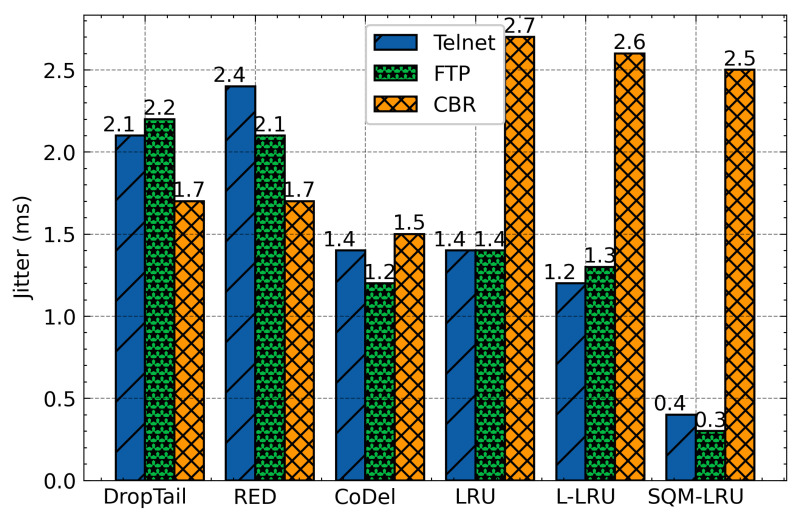
Average jitter comparison.

**Figure 12:**
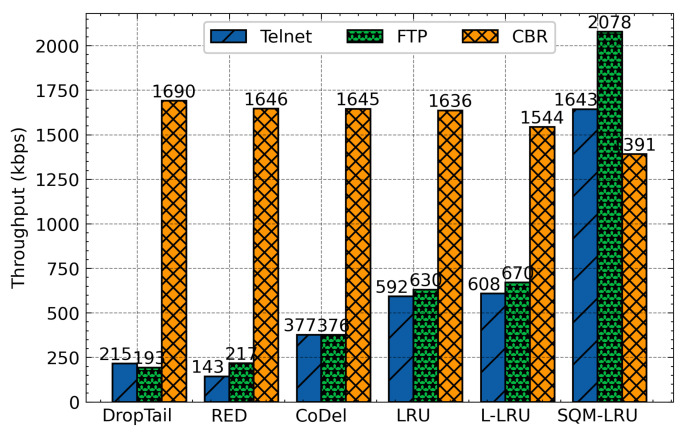
Average throughput comparison.

**Figure 13:**
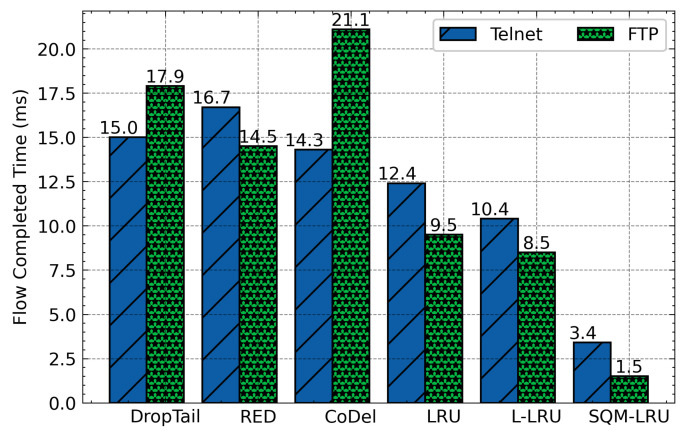
Comparison of average FCT.

The averages delay of these algorithms was presented in Figure 10. According to this figure, as we have expected, DropTail, RED, and CoDel algorithm unable to distinguish flows and achieve fairness, so the delay of the responsive flow is large. The LRU and L-LRU schemes by constructing LRU cache to identify flows and then integrate with the RED to active queue management. However, the flow detection effect is limited, and single-queue is hard to achieve differentiated service requirements. The SQM-LRU adopts SQ and M-LRU for efficient flow identification, and the priority queues are used to achieve fairness and provide differentiated services. Therefore, the delay of responsive flow under SQM-LRU is reduced by about 57%, 55%, 20%, 30%, and 28%, respectively, when compared with DropTail, RED, CoDel, LRU, and L-LRU. Figure 11 indicates the jitter of responsive flow for each queue management algorithm. SQM-LRU can detect the responsive flow and put it into the high-priority sub-queue and using CoDel for queue management, so the performance is guaranteed. Therefore, the jitter of SQM-LRU is reduced by approximately 80%, 83%, 71%, 70%, and 66%, respectively. This is because the other algorithms cannot achieve fairness and differentiated services. Figure 12 further illustrates that under other algorithms, the responsive flow can only obtain extremely low bandwidth due to poor fairness. With SQM-LRU, the throughput of responsive flow increased by 2 to 3×, respectively. Figure 13 shows the FCT of responsive flow was also reduced by about 70% to 85%. The delay and jitter of non-responsive LTF flow (CBR) are increased, and the throughput is reduced, but the decline is slight by approximately 20%, which has little effect on its performance. The above experiments show that the proposed SQM-LRU algorithm can achieve better fairness and help the responsive flow reduce the delay and jitter while maximizing the link utilization of non-responsive LTF flow to ensure their performance requirements.

### 4.3. Varied Number of Flows

**Figure 14:**
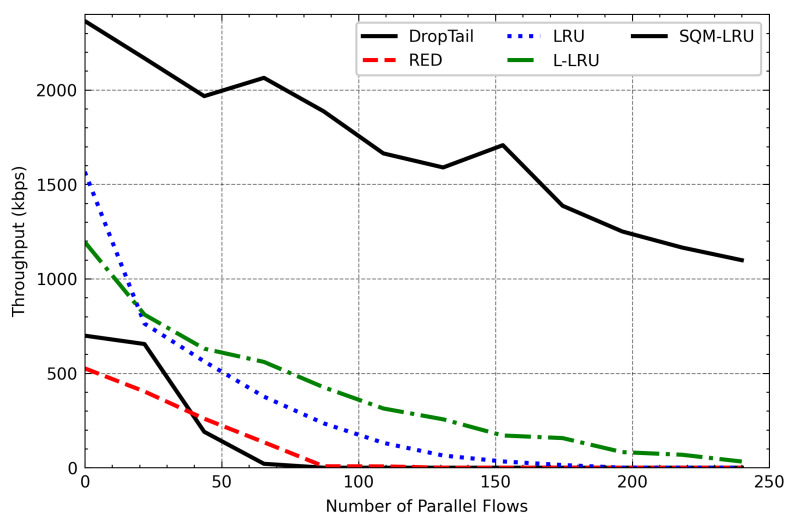
FTP throughput varies with the number of parallel flows.

**Figure 15:**
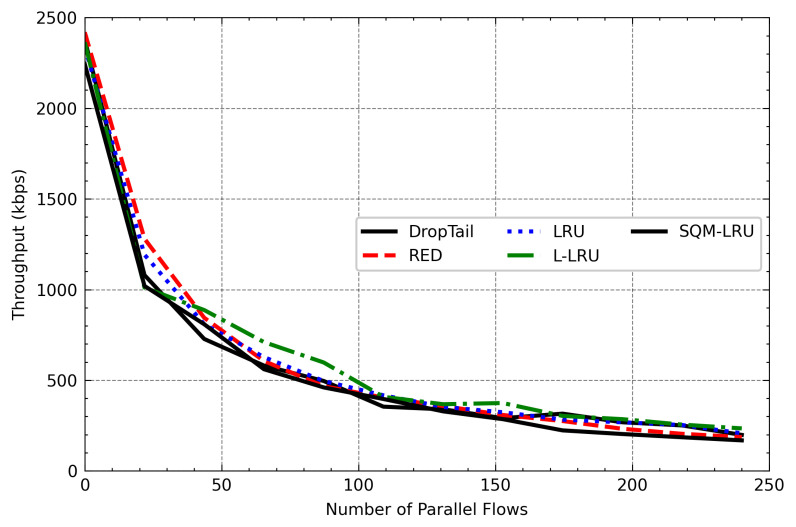
CBR throughput varies with the number of parallel flows.

To check the SQM-LRU performance under different loads, we change the flow number from 20 to 240. When parallel flow increases, the network congestion also increases significantly. Figure 14 shows a detailed view of the various queue management algorithms on the throughput of responsive flow (FTP) across the various parallel flow. On network congestion, non-responsive LTF flow is more competitive and occupies huge network resources. However, DropTail and RED algorithms fail to achieve fairness. As the number of parallel flows increases, the responsive flow throughput decreases significantly. The LRU and L-LRU algorithms adopt a cache to detect different flows, and the throughput of responsive flow is improved compared to DropTail and RED. Because LRU and L-LRU require a larger space to achieve a higher flow detection, the performance is reduced when the number of parallel flows increases. SQM-LRU adopts the SQ and M-LRU to achieve better flow detection. Besides, the responsive flow is enqueued in the high priority sub-queue to ensure its transmission. As a result, its throughput has increased compared to other algorithms. Figure 15 presents the average throughput of the non-responsive LTF flow (CBR) in changing load. SQM-LRU enqueued the non-responsive LTF flow into the low-priority sub-queue, and the low-priority sub-queue adopts the RED algorithm to make full use of link resources, while the responsive flow is small and does not damage to non-responsive LTF flow. Therefore, with the number of parallel flow increases, the average throughput of CBR decreases, and SQM-LRU presents a similar trend compared to other algorithms.

**Figure 16:**
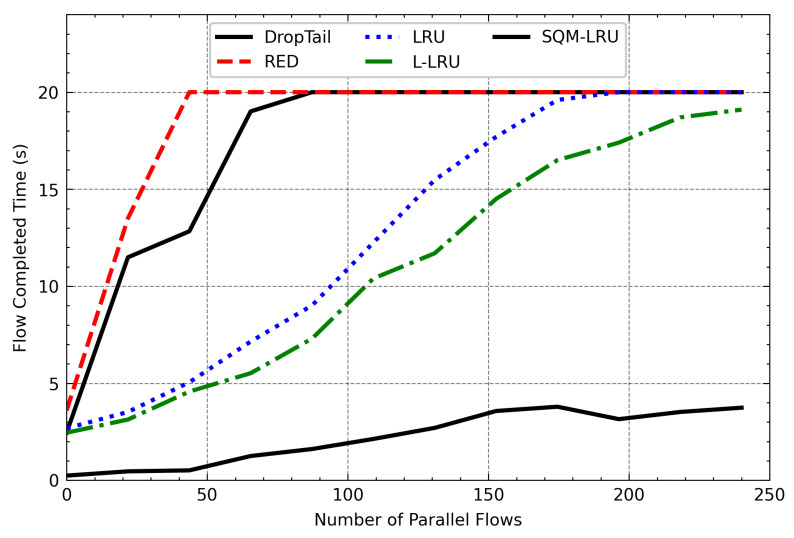
The FCT of FTP varies with the number of parallel flows.

**Figure 17:**
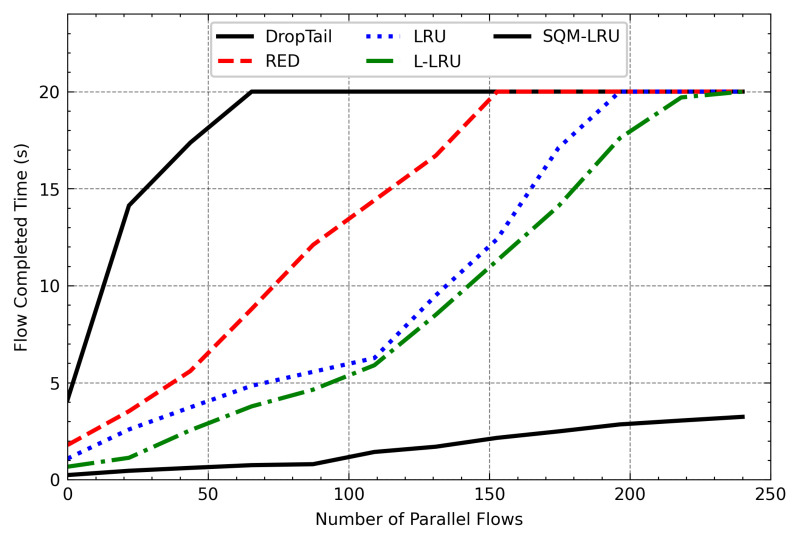
The FCT of Telnet varies with the number of parallel flows.

Figure 16 to Figure 17 provide the overall results of the FCT of FTP and Telnet. It can be seen that when the number of the parallel flows is less, the FCT of those algorithms is closer. As it increases, the FCT of DropTail, RED, LRU, and L-LRU will increase rapidly. Even with the number of parallel flows still increasing, the FTP and Telnet flows cannot be carried out normally. The reason is that FTP and Telnet are the typical responsive flow, DropTail and RED unable to distinguish them. Therefore, the responsive flow can only obtain limited network resources on network congestion, and it takes a long time to complete the transfer. The LRU and L-LRU detect responsive flow when there are fewer parallel flows, but the detection effect gets poor when the parallel flow increases. SQM-LRU detects the responsive flow more efficiently and puts them into high priority sub-queue to guarantee the transmission so that the FCT could be kept low and stable. This result shows that SQM-LRU can achieve better network performance under different loads.

In the traditional queue management algorithms such as DropTail and RED, the JFI is low and poor in fairness, and the unfairness is more significant during network congestion. With parallel flows increase, there is a significant drop in JFI under LRU and L-LRU. In SQM-LRU, the JFI remains at approximately 0.75 at network congestion, indicating SQM-LRU can maintain the fairness between the responsive and non-responsive LTF flow under varying scenarios. The above experiments show that precise flow detection can effectively improve network performance. The parameters setting of the flow detection will be detailed in the following experiments.

### 4.4. Different Shadow Queue Length and Pre-Sampling Probability

**Figure 18:**
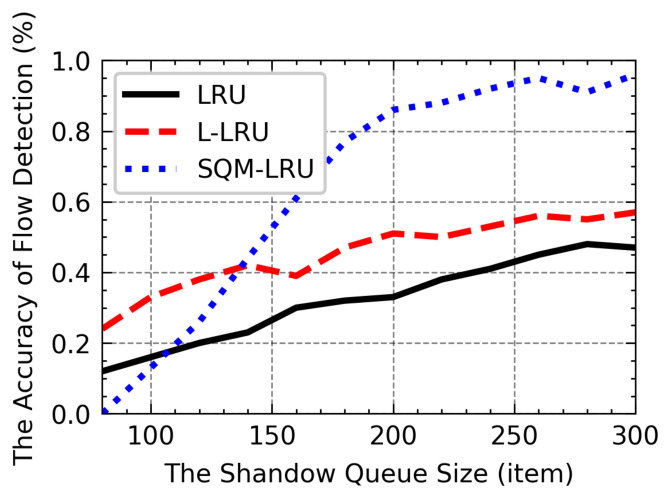
The influence of SQ length on Acc.

**Figure 19:**
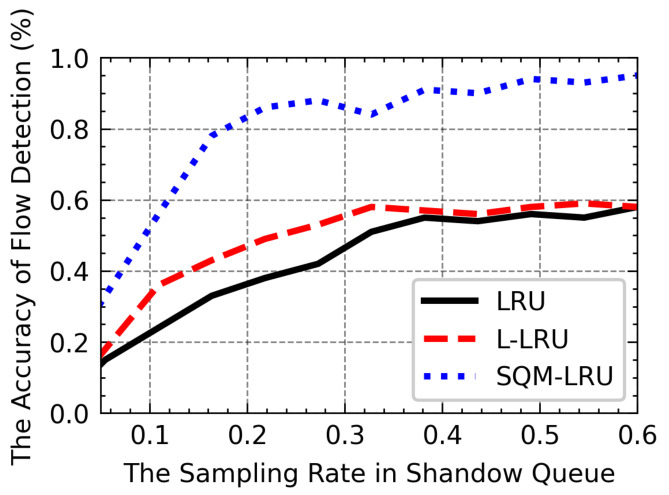
The influence of $p_{samp}$ on Acc.

The following experiments investigate the efficiency of the SQ lengths and pre-sampling probability. As shown in Figure 18, with the increases in SQ length, the LRU-based algorithm can record more historical flow information, and the efficiency of flow detection increases. Under the LRU and L-LRU, the non-responsive LTF flow is frequently replaced due to a large number of responsive flows that lead to it cannot be reported correctly, and the Acc only has a little gain. In SQM-LRU, the use of pre-sampling and the separation mechanism helps to achieve a higher Acc is higher than LRU and L-LRU. When the SQ length reaches 200 or above, the most non-responsive LTF flows are reported correctly, and this may help the responsive flow obtains low delay, etc. Therefore, we select 200 as the most appropriate SQ length setting. It can be seen from Figure 19 with the sampling probability $p_{samp}$ increased, and the sampling results can reflect the distribution of packets which leads the Acc to grow. When the sampling probability approaches 0.5, the $p_{samp}$ improves the Acc slowly but leads to substantial computational load, so the proper sampling probability $p_{samp}$ is set to 0.3–0.5.

### 4.5. The Influence of M-LRU Size

**Figure 20:**
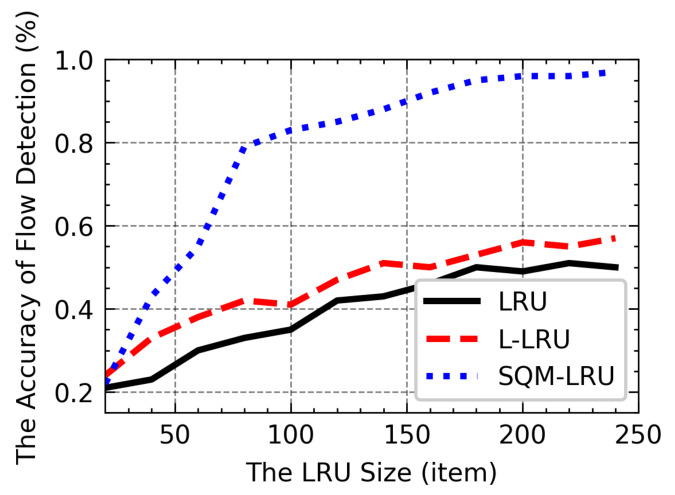
The influence of LRU size on Acc.

Figure 20 shows the effects of having a growing size of LRU on Acc. As the LRU size increases, a greater historical flow could be recorded, which helps to improve flow detection. However, as the non-responsive LTF flow will be frequently replaced by the responsive flow, which results in it cannot be reported correctly, the LRU and L-LRU schemes are poor in flow detection. M-LRU adopts pre-sampling and MARK node to separate the responsive and non-responsive LTF flow units, effectively avoiding the decrease of accuracy caused by the frequent replacement of non-responsive LTF flow in the cache. The detection of non-responsive LTF flows is optimal when the size of the M-LRU is set to ~80. In addition, by adjusting the size of the responsive and non-responsive flow units, the M-LRU’s ability to detect different flows could be further enhanced. Compared to the LRU and the L-LRU, a larger size (~400) is required to ensure accuracy, and the SQM-LRU scheme has a significant improvement in space and accuracy.

### 4.6. Comparison under Different Threshold $p_{lth}$

When the packet count in the M-LRU is greater than the threshold $p_{lth}$, the flow will be reported as a non-responsive LTF flow. A similar experiment was repeated by varying the threshold $p_{lth}$ from 1 to 10. The $p_{lth}$ determines the limit beyond which we start penalizing a flow. A flow can send data and accumulate a count to threshold $p_{lth}$, where it is small, and still get away unpunished if it is able to get out of the cache. The larger of threshold $p_{lth}$, the burstier the flows can be without getting penalized. In short, if the $p_{lth}$ is too small, more responsive flows will be reported as non-responsive LTF flow, and put these flows into the low-priority sub-queue where they will be severely penalized. If the threshold is too large, the non-responsive LTF flow will not be reported accurately and then placed into the high priority sub-queue, which fails to control them, thereby severely affecting the performance of the responsive flow.

**Figure 21:**
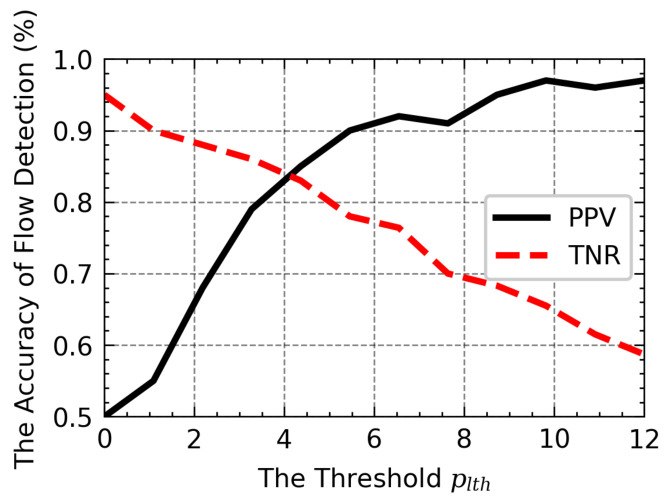
The influence of threshold $p_{lth}$.

A graphical representation of the detection performance of $p_{lth}$ is shown in Figure 21. When the threshold gets smaller, the TNR is more extensive, and the PPV is smaller, which indicates that more responsive flows are incorrectly reported as non-responsive LTF flow. As the threshold increases, the PPV increases, while the TNR decreases. Meanwhile, the non-responsive LTF flow may be reported as responsive flow. Hence, to ensure the flow detection, we choose about 5 as the default threshold $p_{lth}$, which better affects the PPV and TNR, enhances the classifier’s performance to detect those flows.

### 4.7. Queue Management and Scheduling

**Figure 22:**
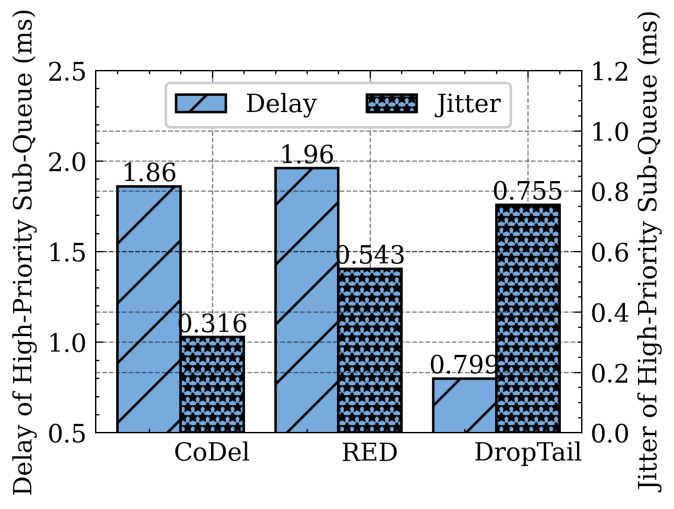
Different high-priority sub-queue management algorithm.

**Figure 23:**
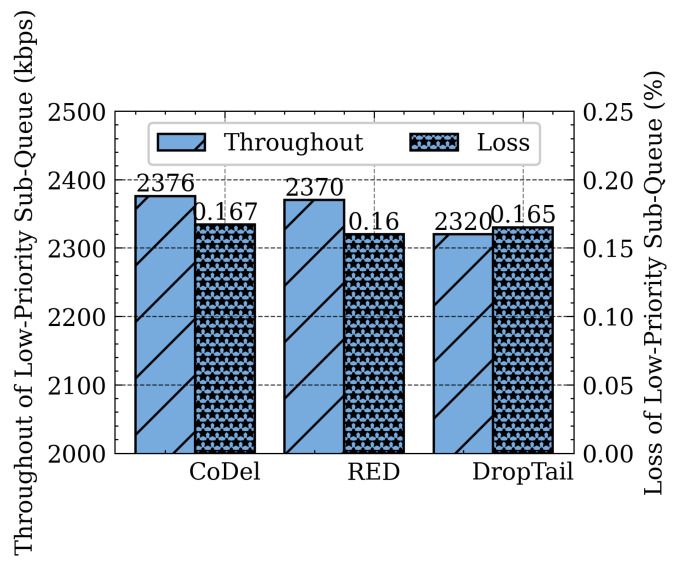
Different low priority sub-queue management algorithm.

The following series of experiments clarifies the impact of different sub-queue management schemes, such as DropTail, RED, and CoDel on flow performance. The delay and jitter of high-priority sub-queue packets when using DropTail, RED, and CoDel are shown in Figure 22. The responsive flow is placed into the high-priority sub-queue, and it requires lower delay and jitter. CoDel can achieve lower delay and jitter than DropTail and RED, so SQM-LRU employs CoDel for the high-priority sub-queue management. Figure 23 shows the throughput of low-priority sub-queue when using CoDel, RED, and DropTail. CoDel and DropTail result in higher packet loss when the network get congested, while RED drops packets with the probability based on queue length while achieving higher throughput. The non-responsive LTF flow desires to maximize the bandwidth without excessive packet loss. Compared with DropTail and CoDel, RED can obtain higher throughput and lower packet loss. Therefore, RED is adopted as the low priority sub-queue management.

**Figure 24:**
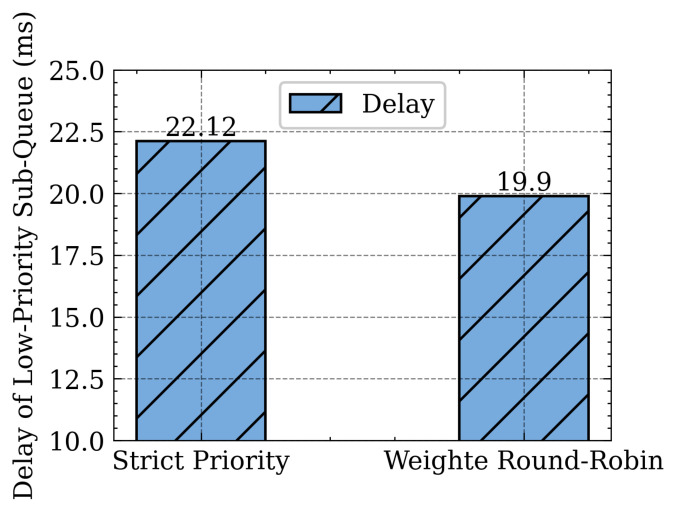
Influence of queue scheduling algorithm.

We compare the SP and WRR Scheduling on the delay of the low priority sub-queue. The low priority sub-queue packet is sent only when the high priority sub-queue is empty in the SP algorithm, which results in a high delay of the low priority sub-queue, as can be seen from Figure 24. The WRR schedules packets between high and low priority sub-queues based on their weights, which effectively reduces the delay of low priority sub-queues and prevents it from “starvation” without being served for a long time. Other more complex queue scheduling algorithms may further enhance performance. Due to space limitations, this paper will not cover the detailed analysis of various scheduling algorithms.

## 5. Conclusions and Future Work

In this paper, the SQM-LRU is proposed, which supports the following: (a) SQM-LRU pre-sampling in SQ and further comparing in M-LRU to detect different flows with low-cost implementation and O(1) time consumption. (b) It adopts the priority dual-queue scheme to put the responsive flow into the high priority sub-queue and the non-responsive LTF flow into the low-priority sub-queue for achieving fairness when those flows coexist. (c) The high- and low-priority sub-queues adopt CoDel and RED, respectively, to achieve low delay, jitter and FCT for responsive flows while maximizing the throughput for non-responsive LTF flows. The simulation results show that the proposed SQM-LRU scheme significantly improves the key performance indicators such as delay, jitter, FCT, etc., compared to the traditional schemes. Benefiting from the low space and time consumption, it is practically deployable. Our next step is to extend the SQM-LRU with a more fine-grained approach to managing queues for various service demands to meet the differentiated QoS requirements and further enhance the overall network performance.

## 6. Patents

The authors of this work have applied for a patent and that patent is undergoing substantive examination.

## Figures and Tables

**Table 1:** Experimental default parameters.

Parameters	Value
SQ Size	200 pkts
$p_{samp}$	30%
$p_{lth}$	5
M-LRU Size	50 items
Non-Responsive LTF Flow Unit Size	20 items
Responsive Flow Unit Size	30 items
Queue Size	100 pkts
Packet Size	1000 bits
Standard TCP	Cubic
TCP Window	20
CBR Rate	3 Mbps

**Table 2:** Confusion matrix.

	Non-Responsive LTF	Responsive Flow
**Non-Responsive LTF**	TP	FN
**Responsive Flow**	FP	TN

## Data Availability

Not applicable.
